# First record of *Perkinsus beihaiensis* in cultured mussels *Mytilus coruscus* in the East China Sea

**DOI:** 10.1017/S0031182024000702

**Published:** 2024-09

**Authors:** Jia Y. Zhai, Peng Z. Qi, Xiao A. Yang, Zhong J. Ren, Zi H. Zhang, Jia X. Gao, Deng H. Zhu, Pei P. Fu

**Affiliations:** 1 National Engineering Research Center of Marine Facilities Aquaculture, Marine Science and Technology College, Zhejiang Ocean University, Zhoushan, Zhejiang, P. R. China; 2 Taizhou Key Laboratory of Biomedicine and Advanced Dosage Forms, School of Life Sciences, Taizhou University, Taizhou, Zhejiang, P. R. China

**Keywords:** *Perkinsus beihaiensis*, *Mytilus coruscus*, ARFTM, ITS, East China Sea

## Abstract

During the investigation of parasitic pathogens of *Mytilus coruscus*, infection of a *Perkinsus*-like protozoan parasite was detected by alternative Ray's Fluid Thioglycolate Medium (ARFTM). The diameter of hypnospores or prezoosporangia was 8–27 (15.6 ± 4.0, *n* = 111) μm. The prevalence of the *Perkinsus*-like species in *M. coruscus* was 25 and 12.5% using ARFTM and PCR, respectively. The ITS1-5.8S-ITS2 fragments amplified by PCR assay had 100% homology to that of *P. beihaiensis*, suggesting that the protozoan parasite was *P. beihaisensis* and *M. coruscus* was its new host in East China Sea (ECS). Histological analysis showed the presence of trophozoites of *P. beihaiensis* in gill, mantle and visceral mass, and the schizonts only found in visceral mass. *Perkinsus beihaiensis* infection led to inflammatory reaction of hemocyte and the destruction of digestive tubules in visceral mass, which had negative effect on health of the farmed *M. coruscus* and it deserves more attention.

## Graphical abstract

**Figure:**
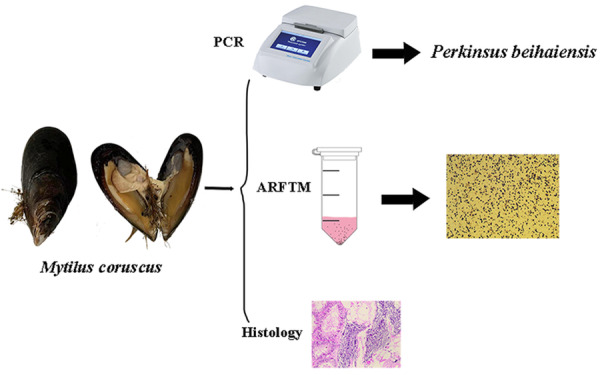

## Introduction

Perkinsosis is the most severe disease in marine mollusks caused by protozoan endoparasites of the genus *Perkinsus* Machkin, 1950. The genus *Perkinsus* belongs to the phylum Perkinsozoa and comprises seven nominal species: *Perkinsus marinus* (Ray, 1996), *Perkinsus olseni* (Lester and Davis, 1981), *Perkinsus qugwadi* (Blackbourn *et al*., 1998), *Perkinsus chesapeaki* (McLaughlin *et al*., 2000), *Perkinsus mediterraneus* (Casas *et al*., 2004), *Perkinsus honshuensis* (Dungan and Reece, 2006) and *Perkinsus beihaiensis* (Moss *et al*., 2008). Since its discovery in 1950, *Perkinsus* spp. have been detected in mollusks around the world, such as in North America, South America, Europe, Asia, Oceania and Africa (Lester and Davis, 1981; Ray, 1996; Casas *et al*., 2004; Moss *et al*., 2008). *Perkinsus marinus* (syn. *Dermocystidium marinum*) is the causative agent of Dermo disease, which leads to seasonal mortality and a decline in the population of the eastern oyster, *Crassostrea virginica*, along the Gulf of Mexico coast of the United States (Ray, 1996). *Perkinsus olseni* has a wide geographical distribution and a wide range of host, including *Haliotis ruber*, *Tapes decussatus*, *Ruditapes philippinarum*, *Crassostrea hongkongensis*, *Pinctada martensii*, *Haliotis laevigata*, as well as several other bivalves and gastropods (Goggin and Lester, 1995; Villalba *et al*., 2004). Owing to the lethal and sublethal impact on commercially valuable mollusks, parasitic disease caused by *P*. *olseni* and *P*. *marinus* have been designated as internationally notifiable mollusk diseases by the World Organization for Animal Health (OIE, 2016).

*Perkinsus beihaiensis* was firstly described in the oysters *Crassostrea ariakensis* and *C. hongkongensis* of Southern China (Moss *et al*., 2008). The reported hosts of *P. beihaiensis* was 15 species of mollusks include oysters, clams (Moss *et al*., 2008; Sanil *et al*., 2012; Ferreira *et al*., 2015; Luz *et al*., 2017; Cui *et al*., 2018; Pagenkopp Lohan *et al*., 2018; Ye *et al*., 2022) and Mediterranean mussel (*Mytilus galloprovincialis*) (Itoh *et al*., 2019). The geographic distribution of *P. beihaiensus* extended to other countries such as India, Brazil, Panama and Japan (Sanil *et al*., 2012; Ferreira *et al*., 2015; Pagenkopp Lohan *et al*., 2018; Itoh *et al*., 2019). Epidemiological investigation showed that the prevalence of *P. beihaiensis* was notably higher during the summer and autumn seasons compared to winter and spring (Yang, 2022).

Over the past decades, the thick shelled mussel *Mytilus coruscus* has emerged as one of the most commercially important shellfish species in China due to its exceptional food quality, significant health benefits and ecological service value (Zhong *et al*., 2014; Liang *et al*., 2015). However, in recent years, the large-scale and high-density cultivation of *M. coruscus*, coupled with changes in climate (such as global warming) and environment factors (such as seawater acidification), has led to various pathogenic challenges to this mussel species (Dong *et al*., 2017). Therefore, it is necessary to investigate the presence of *Perkinsus* species in *M. coruscus* and determine their impacts on health of thick shelled mussels.

## Materials and methods

### Sample collection and process

A total of 16 specimens (Supplementary Table S1) of *M. coruscus* (mean shell length ± s.d.: 9.6 ± 0.6 cm) were sampled in March 2023 from Gouqi island (E122°76′, N30°70′) in Zhoushan city, Zhejiang province, renowned as the hometown of *M. coruscus*. After shucking the shell, a portion of the mantle, gill and digestive gland were fixed in 4% PFA for histopathological examination. Another portion of the gill tissue was fixed in anhydrous ethanol for DNA extraction. The remaining soft body was weighed, shredded and subjected to ARFTM for quantifying the infection level of *Perkinsus* species.

### Detection of *Perkinsus* species in ARFTM

Fifteen mL of ARFTM supplemented with antibiotics (chloramphenicol 200 μg mL^−1^, penicillin-streptomycin 500 U mL^−1^) and nystatin (4.6 μg mL^−1^) was added into each sample tube. After a week of incubation in the dark at room temperature (La Peyre *et al*., 2003). The tissues were collected by centrifugation and then subjected to treatment with 2 M NaOH at 60°C until complete lysis (Choi *et al*., 1989). Hypnospores or prezoosporangia were subsequently collected by centrifugation (4000 rpm, 20 min), followed by three washes with sterile seawater and fixed volume to 1 mL. Eighty μL of hypnospores suspension thorough mixed with 20 μL Lugol's iodine, then 10 μL of this mixture was transferred onto a hemocytometer to count the number of hypnospores with a bluish black spherical shape. Each sample underwent three counting repetitions.

The prevalence of infection by *Perkinsus* was expressed as the number of infected animals over the total number of sampled animals (Bush *et al*., 1997), while the infection intensity was calculated as the number of *Perkinsus* cells per gram of tissue weight (Park and Choi, 2001).

### Histological observation

The fixed tissues were dehydrated with aqueous ethanol through an ascending series of concentrations, cleared with xylene, embedded in paraffin and 6 μm thick sections were made using a Microtome (Leica, Germany). The sections were stained with hematoxylin & eosin, then mounted in Canada balsam for observation under a light microscope compound with brightfield optics (Leica, Germany).

### DNA extraction, PCRs, sequencing and phylogenetic analysis

Genomic DNA was extracted from excised gill using a TIAN amp Genomic DNA Kit (TIANGEN, Beijing, China), following the manufacturer's protocols. The *Perkinsus* genus-specific internal transcribed spacer (ITS) ribosomal RNA primers (PerkITS-85: 5′-CCGCTTTGTTTGGATCCC-3′; PerkITS-750: 5′-ACATCAGGCCTTCTAATGA TG-3′) were applied to amplify the target sequence (Casas *et al*., 2002). The final volume of the polymerase chain reaction (PCR) was 20 μL containing 10 μL of 2xEs Taq Master Mix (Hangzhou, China), 10 pmol of each PCR primer, and 1 μL of the extracted DNA samples. The thermal cycler programme was as follows: 5 min at 94°C as the initial step; then 35 cycles of 30 s at 94°C, 45 s at 56°C, 45 s at 72°C. The final step was 7 min at 72°C. All PCR products were loaded on a 1.5% agarose gel. After purification, the positive PCR products were sequenced using the both PCR primers on an ABI 3730XL (Applied Biosystems, USA). The sequences were assembled manually with the software SeqMan (DNASTAR, USA). The homology of the generated sequences was analysed using the Basic Local Alignment Search Tool (BLAST) program available on the NCBI.

The maximum likelihood (ML) method was employed to construct phylogenetic trees using ITS sequences in IQ-tree (Nguyen *et al*., 2014). Based on the Bayesian information criterion (BIC), HKY + F + G4 was chosen as the optimal nucleotide substitution model for ITS in PhyloSuite (Zhang *et al*., 2020). ML trees were obtained with 1000 bootstraps of the data.

## Results

### Identification of *Perkinsus* species in *M. coruscus*

**Figure 1.**
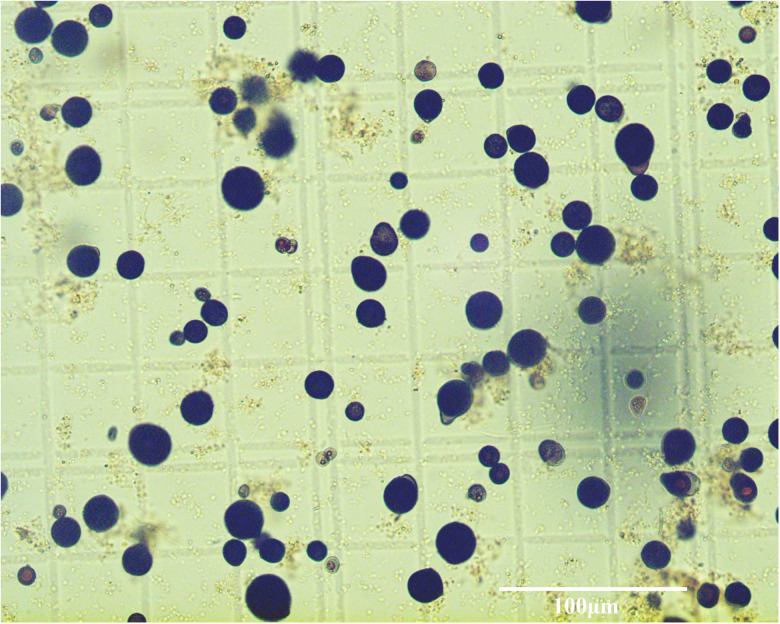
The hypnospores or prezoosporangia of *Perkinsus beihaiensis* in *Mytilus coruscus*. Scale bars = 100 μm.

According to the ARFTM assay, four positive samples exhibited enlarged blue–black hypnospores characteristic of *Perkinsus* sp. like organisms, and the prevalence of infection by *Perkinsus* sp. like organisms in *M. coruscus* was 25% (4/16). The diameter of hypnospores or prezoosporangia was 8–27 (15.6 ± 4.0, *n* = 111) μm (Fig. 1). The infection intensity ranged between 10.2 and 3.34 × 10^5^ cells per gram tissue. However, there were only two positive samples in the PCR assay, and the prevalence of infection was 12.5% (2/16). The PCR primers targeting the ITS region of *Perkinsus* spp. amplified 687 bp of products. Sequence alignment found that the two positive samples had the same ITS sequences, and the BLAST results revealed that the sequence had the highest homology with *P*. *beihaiensis* (Taizhou, China, accession number: MT908890) at 100. The nucleotide sequence obtained in the current study has been deposited in NCBI GenBank with accession numbers OR192847 and OR192848.

### Phylogenetic analysis

**Figure 2.**
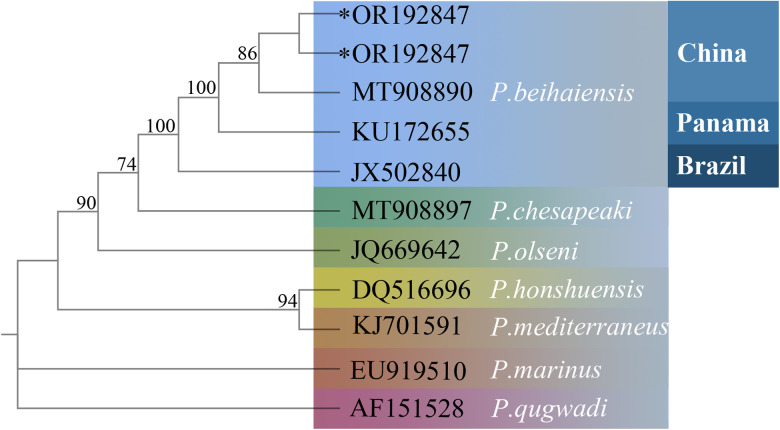
Phylogenetic relationships of *Perkinsus* species based on ITS sequences constructed by ML method. ‘*’ represented the sequences obtained in this study.

The ML phylogenetic tree (Fig. 2) indicated that the specimens of *Perkinsus* from Gouqi island cluster with *P. beihaiensis* from China, Panama and Brazil. Internal transcribed spacer region nucleotide sequences of *P. beihaiensis*, *P. chesapeaki*, *P. olseni* formed a monophyletic clade sister to a clade containing *P. honshuensis* and *P. mediterraneus*.

### Histological observations

**Figure 3.**
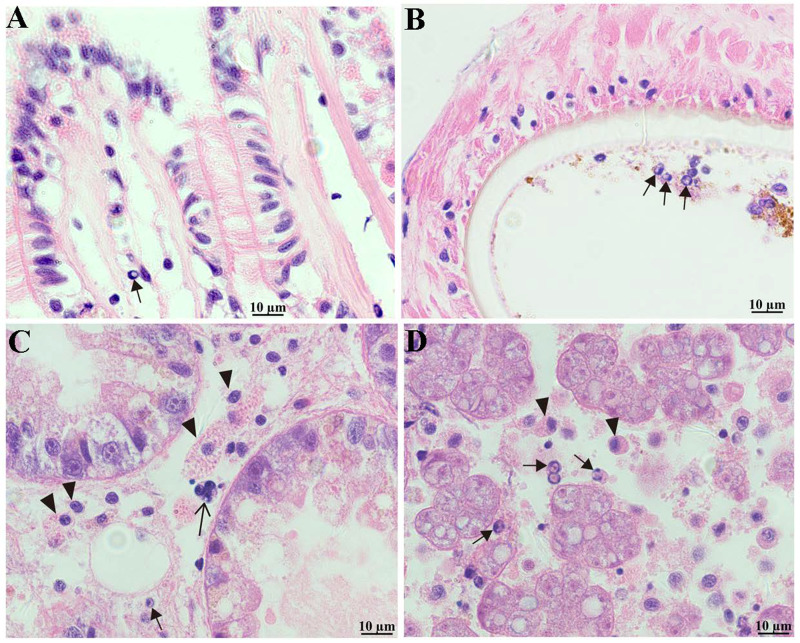
*Perkinsus beihaiensis* infection in the gill (A), mantle (B) and visceral mass (C) of *M*. *coruscus*. (↑) and (▵) represents the trophozoite and schizont of the *P. beihaiensis*. (▴) represent the hemocyte of *M. coruscus*. Scale bars = 10 μm.

*P*erkinsus *beihaiensis* was observed in the visceral mass, gills and mantle (Fig. 3). The mature trophozoites, characterized by a large vacuolation and eccentric nucleus, were found in the three tissues, especially in visceral masses (Fig. 3A, B, D), and the schizonts were mainly observed in visceral mass (Fig. 3C). The infection of *P. beihaiensis* induced the hemocyte infiltration in the visceral mass (Fig. 3C, D). Although no apparent lesions were observed in the tissues, destruction of digestive tubules was evident (Fig. 3C, D).

## Discussion

*Perkinsus beihaiensis,* a cosmopolitan parasite species, has a wide geographical distribution, including Asia (China, Japan and India), South America (Brizal and Panama) (Moss *et al*., 2008; Sanil *et al*., 2012; Pagenkopp Lohan *et al*., 2018; Itoh *et al*., 2019; Ye *et al*., 2022). In China, the main epidemic area of *P. beihaiensis* was in SCS (Moss *et al*., 2008; Cui *et al*., 2018; Wu *et al*., 2018), and recently been reported in ECS and YBS (Ye *et al*., 2022). Although in previous study, *P. beihaiensis* has been detected in ESC, including Taizhou (Zhejiang province), Ningde and Putian (Fujian province) (Ye *et al*., 2022). However, the sample of collection location in the presently study was in Shengsi county (Zhoushan, Zhejiang province), the main production area of *M. coruscus*, that has not been investigated for *Perkinsus* species detection in ESC.

*Perkinsus beihaiensis* was firstly detected in a new host *M. coruscus* through ARFTM, histological observation and PCR in this study, expanding the host range of the parasite. The host specificity of *P. beihaiensis* is low (Ye *et al*., 2022). It has so far been reported in 15 bivalve species (Pagenkopp Lohan *et al*., 2018; Itoh *et al*., 2019), and clams and oysters are the known susceptible hosts of the parasite. *Perkinsus beihaiensis* was the dominant *Perkinsus* species in the Mediterranean mussel (Itoh *et al*., 2019). Consistently, *P. beihaiensis* was the only *Perkinsus* species detected from *M. coruscus*, a congeneric species of *M. galloprovincialis*, in the present study, indicating that mussel seems to be highly susceptible to this parasite.

*Mytilus coruscus* is mainly distributed in the Hokkaido of Japan, Jeju Island of Korea, the Yellow Sea, Bohai, the East China Sea (ECS) and Taiwan (Dong *et al*., 2017). With the breakthrough of artificial seedling cultivation technology, the breeding scale of *M. coruscus* has increased rapidly. It has become one of the most important native economic species in China recently with outstanding economic value. The cultivation of this mussel is raft farming charactered with a high degree of intensification and breeding density, which may provide conditions for the widespread of *P. beihaiensis* in *M. coruscus*.

Histological tissue section in oysters moderate infected with *P. beihaiensis* induced lesions and hemocyte defensive responses occurring in epithelia and connective tissues (Moss *et al*., 2008). Contrastingly, the pathological effects caused by *P. beihaiensis* in *M. coruscus* appeared to be milder. Infection with this *Perkinsus* species could lead to inflammatory reaction of hemocyte in visceral mass and the destruction of digestive tubules was evident indicating that this parasite had negative physiological effects on *M. coruscus*. Therefore, close attention should be paid to the *P. beihaiensis* in the mussel to prevent harmful effects caused by this parasite to the local aquaculture industry.

## Supporting information

Zhai et al. supplementary material Zhai et al. supplementary material

## Data Availability

Representative sequences obtained in this study were deposited in GenBank with the accession numbers OR192847–OR192848.

## Supplementary material

The supplementary material for this article can be found at https://doi.org/10.1017/S0031182024000702.
