# Supplementary material for: First record of Perkinsus beihaiensis in cultured mussels Mytilus coruscus in the East China Sea
Source: Parasitology. 2024 Nov 21;151(10):1104–7. doi: 10.1017/S0031182024000702 (PMC11894007; doi:10.1017/S0031182024000702)
Supplement: Zhai et al. supplementary material [file S0031182024000702sup001.docx]

**Table S1**. Sample information of *M. coruscus*

| Sample ID | SL (cm) | SW (cm) | ST (cm) | W (g) | SW (g) |
| --- | --- | --- | --- | --- | --- |
| 161 | 9.4 | 4.3 | 3.2 | 57.23 | 16.73 |
| 162 | 10.5 | 4.9 | 2.8 | 64.18 | 18.72 |
| 163 | 10.1 | 5.1 | 2.7 | 63.98 | 20.62 |
| 164 | 9.5 | 4.6 | 3.1 | 57.17 | 18.11 |
| 165 | 10.6 | 5.3 | 3.3 | 71.58 | 23.54 |
| 166 | 9.3 | 4.4 | 3.1 | 56.62 | 17.07 |
| 167 | 9.7 | 4.5 | 3.2 | 63.77 | 19.81 |
| 168 | 9.8 | 4.7 | 2.4 | 57.06 | 19.10 |
| 169 | 9.2 | 4.3 | 2.8 | 47.92 | 14.44 |
| 170 | 9.6 | 4.4 | 3.2 | 59.11 | 18.55 |
| 171 | 9.1 | 4.3 | 2.7 | 56.77 | 19.44 |
| 172 | 10.6 | 5.4 | 2.9 | 73.65 | 22.80 |
| 173 | 9.3 | 4.5 | 3.1 | 55.90 | 16.90 |
| 174 | 9.4 | 4.6 | 2.7 | 52.88 | 17.88 |
| 175 | 8.9 | 4.2 | 2.8 | 50.00 | 15.76 |
| 176 | 8.5 | 4.3 | 2.3 | 35.40 | 9.85 |

Abbreviation:

SL: shell length; SW: shell width; ST: shell thickness; W: weight; SA: sample weight.
